# Streaming MASSIF: Cascading Reasoning for Efficient Processing of IoT Data Streams

**DOI:** 10.3390/s18113832

**Published:** 2018-11-08

**Authors:** Pieter Bonte, Riccardo Tommasini, Emanuele Della Valle, Filip De Turck, Femke Ongenae

**Affiliations:** 1 IDLab, Department of Information Technology, Ghent University—IMEC, B-9052 Ghent, Belgium; filip.deturck@ugent.be (F.D.T.); femke.ongenae@ugent.be (F.O.); 2 Politecnico di Milano, Department of Electronic, Informatics and Bioengineering, 20133 Milan, Italy; riccardo.tommasini@polimi.it (R.T.); emanuele.dellavalle@polimi.it (E.D.V.)

**Keywords:** Stream Reasoning, complex event processing, description logic reasoning, Cascading Reasoning, IoT

## Abstract

In the Internet of Things (IoT), multiple sensors and devices are generating heterogeneous streams of data. To perform meaningful analysis over multiple of these streams, stream processing needs to support expressive reasoning capabilities to infer implicit facts and temporal reasoning to capture temporal dependencies. However, current approaches cannot perform the required reasoning expressivity while detecting time dependencies over high frequency data streams. There is still a mismatch between the complexity of processing and the rate data is produced in volatile domains. Therefore, we introduce Streaming MASSIF, a Cascading Reasoning approach performing expressive reasoning and complex event processing over high velocity streams. Cascading Reasoning is a vision that solves the problem of expressive reasoning over high frequency streams by introducing a hierarchical approach consisting of multiple layers. Each layer minimizes the processed data and increases the complexity of the data processing. Cascading Reasoning is a vision that has not been fully realized. Streaming MASSIF is a layered approach allowing IoT service to subscribe to high-level and temporal dependent concepts in volatile data streams. We show that Streaming MASSIF is able to handle high velocity streams up to hundreds of events per second, in combination with expressive reasoning and complex event processing. Streaming MASSIF realizes the Cascading Reasoning vision and is able to combine high expressive reasoning with high throughput of processing. Furthermore, we formalize semantically how the different layers in our Cascading Reasoning Approach collaborate.

## 1. Introduction

Due to the rise of the Internet of Things (IoT) and the popularity of Social Media, huge amounts of frequently changing data are continuously produced [1,2]. This data can be considered as unbounded streams. In order to extract meaningful insights from these streams, they should be combined and integrated with background knowledge [3]. For example, in the Smart City of Aarhus [4], sensors have been integrated into multiple aspects of the city: traffic sensors to measure the traffic density, sensors to capture the occupation of parking spots, and pollution sensors to measure the pollution values over the city. Since the sensory data typically only describe the sensor readings, it needs to be combined with additional data, e.g., the type of measurement linked to the sensor and the location of the sensor. Combining streams and integrating background knowledge introduces more context and ensures more accurate results. Semantic Web technologies proved to be an ideal tool to fulfill these requirements [5,6,7]. Ontology languages, such as Web Ontology Language (OWL), allow one to model a certain domain and formally specify its domain knowledge. Expressive reasoning and Complex Event Processing (CEP) techniques allow one to extract implicit facts from the streams, enabling meaningful analysis [8,9,10]. Expressive reasoning, such as Description Logic (DL) reasoning [11], which can be used to reason about ontology models, allow one to infer implicit facts conform to the domain knowledge defined in the model. We focus on DL reasoning, as it is a web standard and widely adopted.

For example, a street can be considered to be a ‘high traffic street’ when there have been at least two high traffic observations and each type of street has other thresholds and requirements in order to accurately label an observation as a high traffic observation. In order to accurately interpret the traffic stream, a well-defined background model is necessary. The more accurately one wants to define its domain, the more expressive the required reasoning has to be to correctly interpret the domain. However, higher expressivity of reasoning requires higher complexity of processing [9]. If we want to detect decreasing levels of traffic, we need to detect a temporal relation between low traffic observations and high traffic observations. More specifically, we need to detect when high traffic observations are followed by low traffic observations within a certain amount of time. Furthermore, we need to be able to filter out only those traffic updates going from high to low occurring in the same location.

RDF Stream processors (RSPs) [12,13,14] tackle the problem of combining various streams, integrating background knowledge and processing the data. They focus on efficiency of processing streams and only allow low expressive reasoning or no reasoning at all. Existing work on expressive DL reasoning has focused on static [15] or slowly changing [16] data. The problem of performing expressive reasoning over high velocity streams is, however, still not resolved [17]. Furthermore, temporal DL tends to become easily undecidable [18], making it even harder to perform temporal reasoning over high velocity streams. Through the use of CEP engines, temporal dependencies can be defined in various patterns.

However, CEP engines struggle to integrate complex domains, which makes it difficult to define complex patterns [10].

Stuckenschmidt et al. [9] envisioned the possibility to trade off complexity of processing and data change frequency in order to perform expressive reasoning over high velocity streams. They named this vision Cascading Reasoning, presenting various layers of processing, each with different complexities. To the best of our knowledge, this vision inspired several RSP works, but this paper reports the first attempt to realize the vision and offers blueprints for practitioners willing to exploit it in alternative implementations.

To allow the development of services that can provide intelligent decision making based on heterogeneous streaming data, we set the following *objectives*:

1. *Combine various data streams*: To make meaningful analysis we need to combine streams from various sensors.
2. *Integrate background knowledge*: Since the sensory data typically only describe the sensor readings, we need to be able to link additional data, e.g., the type of measurement linked to the sensor and the location of the sensor.
3. *Integrate complex domain knowledge*: In order to correctly interpret the domain, domain knowledge needs to be integrated. The more accurate the domain definition, the more complex the domain knowledge and the higher the required reasoning expressivity.
4. *Detect temporal dependencies*: Understanding the temporal domain is often necessary when processing streaming data, as many events in data streams have temporal dependencies.
5. *Easy subscription:* To allow service to subscribe to the data of their interest, they should be able to define their information need in a straightforward and declarative manner.

To tackle the challenges of performing expressive reasoning and detecting temporal dependencies over high velocity streams, we introduce Streaming MASSIF, a layered Cascading Reasoning realization. Streaming MASSIF allows IoT services to subscribe to high-volatile streams using high-level concepts and temporal dependencies, which can be evaluated using expressive reasoning techniques. This allows one to tackle highly complex domains, while keeping the subscription definitions simple. For example, since more and more employees have flexible working hours, we would like to create a service that notifies them when it is a good time to go home. More specifically, that is when traffic near their offices starts decreasing. This notification should only be considered if the office allows flexible working hours. To enable this, multiple streams need to be combined and integrated with background knowledge, complex domain knowledge needs to be considered in order to correctly interpret the observations, and temporal dependencies need to be detected to observe the decrease in traffic.

Our Cascading Reasoning approach combines RDF Stream Processing (RSP), expressive DL reasoning, and CEP, in order to perform expressive and temporal reasoning over high volatile streams. We seamlessly combine DL and CEP, enabling the definition of patterns using high-level concepts. This enables the use of complex domain models within CEP and integrates a temporal notion in DL. The integration of RSP tackles the high velocity aspect of the streams. Furthermore, we introduce a query language that bridges the gap between stream processing, expressive reasoning, and complex event processing. This allows the service to easily define the data they would like to subscribe to. Furthermore, we formalize semantically how the different layers collaborate.

We show that Streaming MASSIF is able to handle expressive reasoning and complex event processing over high velocity streams, up to hundreds of events per second.

The paper is structured as follows: Section 2 describes the related work. Section 3 describes all the required background knowledge to understand the remainder of the paper. Section 4 introduces the Streaming MASSIF platform, while Section 5 describes the implications of combining layers more formally. Section 6 details the evaluation of our platform. Section 7 discusses the results, the limitations of the platform, and how our platform compares to the state of the art. The conclusion and our outlook and direction for future work is elaborated in Section 8.

## 2. Related Work

We now elaborate on the related work in the literature and the drawbacks of these previous approaches.

EP-SPARQL [13] is an RSP engine that focuses on event processing over basic graph patterns using Allen’s Algebra for detecting temporal dependencies. However, the reasoning expressivity is low (RDFS) and the definition of event patterns is complex.

StreamRule [19] is a two-layered platform that combines RSP with *Answer Set Programming* (ASP) [20]. However, there is no support for additional layers such as CEP and the two layers are not integrated in a unifying query language for easy usage.

Ali et al. [21] proposed an IoT-enabled communication implemented on StreamRule that performs event-condition-actions rules in ASP. This allows one to define action rules on specific events detected in the stream.

In the CityPulse project [22], the combination of RSP, CEP and expressive reasoning through ASP is presented. The combination of RSP and ASP is supported by StreamRule. In order to handle CEP rules, the system can be extended programmatically, which makes the definition and overview of event patterns complex.

To the best of our knowledge, existing Semantic Complex Event Processing (SCEP) solutions focus on enriching events with semantic technologies.

Teymourian et al. [10] proposed a knowledge-based CEP approach where events are enriched using external knowledge bases. The enrichment is defined using multiple SPARQL queries. However, the system is event-based, there is no support for streaming data, and reasoning is only provided in the external knowledge base that is used for the event enrichment. Thus, no reasoning on the events themselves is possible.

Taylor et al. [23] proposed a SCEP approach that allows one to generalize query definition for CEP engines, enabling interoperability. This is done by defining the event processing operators as ontology concepts. These generalized queries can then be translated into a target language, for example in Event Processing Language (EPL). However, reasoning and streaming data are not taken into account.

Gillani et al. [24] extended the SPARQL query language to include CEP operators. However, reasoning is not taken into account. The benefits of decoupling expressive ontological and temporal reasoning through the use of CEP has been shown in Tommasini et al. [25] and Margara et al. [26].

The MASSIF platform [27] is an event-driven platform IoT platform, allowing service subscription using high-level ontological concepts. MASSIF facilitates the annotation of raw sensor data to semantic data and allows the development and deployment of modular semantic reasoning services which collaborate in order to allow scalable and efficient processing of the annotated data. Each one of the services fulfills a distinct reasoning task and operates on a different ontology model. The Semantic Communication Bus (SCB) facilitates collaboration between services. Services indicate in which types of data they are interested in, referring to high-level ontology concepts. The SCB can coordinate the data on a high-level through the use of semantic reasoning.

Although MASSIF is an event-driven platform, it processes one event at a time and is thus not able to process streams nor capture temporal dependencies between events.

## 3. Background on Cascading Reasoning

In this section, we introduce the necessary knowledge to understand the content of the paper. First, we introduce the original cascading reasoning vision and all the frameworks contained in its layers.

### 3.1. The Original Cascading Reasoning Vision

**Figure 1:**
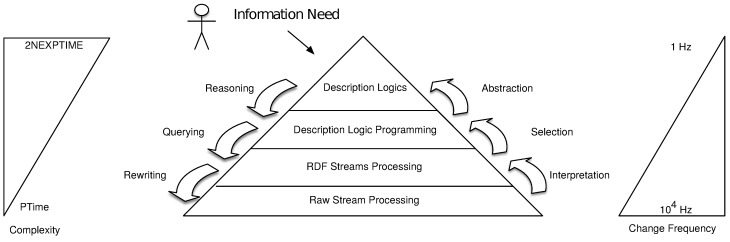
Cascading Reasoning.

Stuckenschmidt et al.’s vision of **Cascading Reasoning** [9] consisted of four layers: *Raw Stream Processing*, *RDF Stream Processing (RSP)*, *Logic Programming (LP)*, and *Description Logics (DL)*, as depicted in Figure 1. Starting from the bottom, each of the layers increases in complexity of processing and reduces the amount of data that is forwarded to the next level. By reducing the data in each layer, higher complexity layers receive fewer data and can still be utilized efficiently.

#### 3.1.1. Raw Stream Processing

This application domain comprises the bottom layer of the Cascading Reasoning pyramid and refers to those systems capable of processing large amounts of information in a timely fashion.

Raw Stream processing or **Information Flow Processing** (IFP) [28] describes how to timely process unbounded sequences of information, also called streams. IFP systems are divided into Data Stream Management Systems (DSMS) and Complex Event Processing (CEP) engines.

DSMSs extend traditional Data Base Management Systems to answer continuous queries that are registered and continuously evaluated over time.

**Figure 2:**
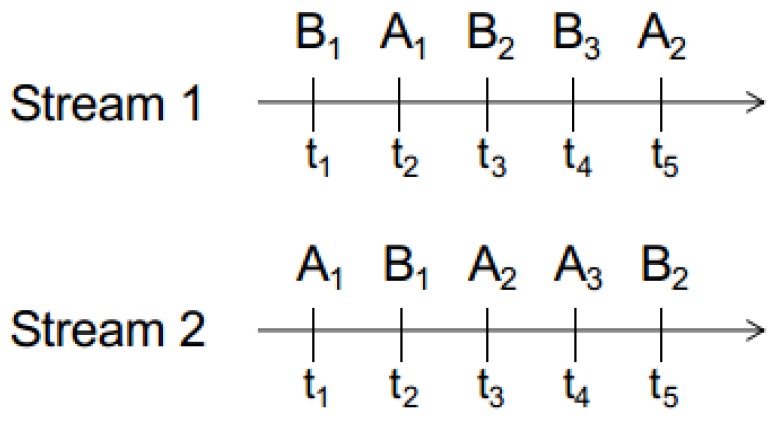
Two example streams to illustrate the various event operators. Each of the streams produces events of the type A or B at different time steps, indicated by $t_{i}$.

CEP Engines [29] are able to capture time dependencies between events. Complex events can be defined through event patterns consisting of various event operators. Examples of these event operators are the time-aware extensions of boolean operators (AND, OR) and the sequencing of events (SEQ). In the following, we present a list of the most prominent CEP operators, guards, and modifiers:

- *AND* is a binary operator: A AND B matches if both A and B occur in the stream and turns true when the latest of the two occurs in the stream. In Figure 2, A AND B matches at $t_{2}$ in both Stream 1 and Stream 2.
- *OR* is a binary operator: A OR B matches if either A or B occurs in the stream. In Figure 2, A OR B matches at $t_{1}$ in both Stream 1 and Stream 2.
- *SEQ* is a binary operator that takes temporal dependencies into account. A SEQ B matches when B occurs after A, in the time-domain. In Figure 2, A SEQ B matches at $t_{3}$ in Stream 1 and at $t_{2}$ in Stream 2.
- *NOT* is a unary operator: NOT A matches when A is not present in the stream. NOT A matches at $t_{1}$ in Stream 1 and $t_{2}$ at Stream 2.
- *WITHIN* is a guard that limits the scope of the pattern within the time domain. A SEQ A WITHIN 2 s matches in Figure 2 at $t_{3}$ in Stream 2 and has no match in Stream 1.
- *EVERY* is a modifier that forces the re-evaluation of a pattern once it has matched. EVERY A SEQ B matches at $t_{3}$ in Stream 1 and at $t_{2}$ & $t_{5}$ in Stream 2 for $\left(A_{2},B_{2}\right)$ and $\left(A_{3},B_{2}\right)$.

For example, we can define a decreasing traffic observation as every high traffic observation followed by a low traffic observation within a certain amount of time, with the following event pattern:

DecreasingTraffic = EVERY HighTraffic SEQ LowTraffic WITHIN 10 m.
However, it is not straightforward in CEP to define what a HighTraffic or LowTraffic exactly is.

For a comprehensive list of operators, we point the reader to Luckham [29]. Note that more advanced temporal relations exist, such as the ones presented in Allen’s interval algebra [30].

#### 3.1.2. RDF Stream Processing

**RDF Stream Processing** (RSP) [3] is an extension of IFP that can cope with heterogeneous data streams by exploiting semantic technologies. Resource Description Framework (RDF) streams are semantically annotated data streams encoded in RDF. RSP-QL [31] is a recent query language formalization that unifies the semantics of the existing approaches with a special emphasis on the operational semantics. In the following, we introduce some of RSP-QL definitions that are relevant to understand the next sections:

**Definition** **1.** *An**RDF Stream**S is a potentially infinite multiset of pairs (G${}_{i}$, t${}_{i}$), with G${}_{i}$ an RDF Graph and t${}_{i}$ a timestamp:*

$$S=\left(g_{1},t_{1}\right),\left(g_{2},t_{2}\right),\left(g_{3},t_{3}\right),\left(g_{4},t_{4}\right),\;\ldots .$$

Since a stream *S* is typically unbounded, a window is defined upon the stream in which the processing takes place.

**Definition** **2.** *A* ***Window*** *W(S) is a multiset of RDF graphs extracted from a stream S. A* ***time-based window*** *is defined through two time instances o and c that are respectively the opening and closing time instants of each window: $W^{\left(o,c\right]}\left(S\right)=\left\{\left(g,t\right)|\left(g,t\right)\in S\wedge t\in \left(o,c\right]\right\}$.*

Note that physical windows, based on the number of triples in the window, also exist [12].

**Definition** **3.** *A* ***time-based sliding window*** *W consumes a stream S and produces a time-varying graph ${\bar{G}}_{W}$. W operates according to the parameters $\left(\alpha ,\beta ,t^{0}\right)$: it starts operating at $t^{0}$, and it has a window width (α) and sliding parameter (β).*

We now introduce the concepts of time-varying graphs and instantaneous graphs. The former captures the evolution of the graph over time, while the latter represents the content of a graph at a fixed time instant.

**Definition** **4.** *A* ***time-varying graph*** *${\bar{G}}_{W}$ is a function that selects an RDF graph for all time instants t∈T where W is defined:*

$${\bar{G}}_{W}:T\to \{G|G_{i}\;an\;RDF\;graph\}.$$

*The RDF graph identified by the time-varying graph, at the time instant t, is called an* ***instantaneous graph*** *${\bar{G}}_{W}\left(t\right)$.*

A dataset used within RSP-QL is defined as follows:

**Definition** **5.** *An* ***RSP-QL dataset*** *SDS is a set consisting of an (optional) default graph and n named graphs describing the static background data and m named time-varying graphs resulting from applying time-based sliding windows over o≤m streams, with m,n≥0.*

**Example** **1.** *In our example, the SDS is defined as*

$$SDS=\{G_{0}=G_{sensors},\left(w_{1},W_{1}\left(S_{traffic_{1}}\right)\right),\left(w_{2},W_{2}\left(S_{traffic_{2}}\right)\right),...\left(w_{n},W_{n}\left(S_{traffic_{n}}\right)\right)\}.$$

*$G_{sensors}$ describe the domain knowledge and the static data about the sensors such as their kinds and their locations. $S_{traffic_{i}}$ describes the traffic observations and is windowed in $W_{i}$. $w_{i}$ is the window name.*

To be able to query the *SDS* dataset, we define an RSP-QL query:

**Definition** **6.** *An* ***RSP-QL query*** *Q is defined as (SE, SDS, ET, QF) where*

- *SE is an RSP-QL algebraic expression;*
- *SDS is an RSP-QL dataset;*
- *ET is a sequence of time instants on which the evaluation of the query occurs;*
- *QF is the Query Form (e.g., Select or Construct)*

#### 3.1.3. Description Logic Programming

The reasoning application domain consists of the top two layers of the Cascading Reasoning pyramid. It refers to systems capable of deriving implicit knowledge from the input data combined with rules and domain models. The first reasoning layer in the original Cascading Reasoning vision was Logic Programs.

**Logic Programs** (LPs) are sets of rules of the form head ← body that can be read as *head “if" body*. The original vision of Cascading Reasoning referred to a specific fragment of LPs, called Description Logic Programs (DLPs) [32], which consists of the intersection between Description Logics and those LPs also expressible in First Order Logics. DLPs can be seen of an ontological sub-language of DL that can be encoded in rules.

#### 3.1.4. Description Logics

The popularity of OWL has led to the design of OWL2, defining the foundations of OWL2 DL reasoning.

**Description Logics** [11], the second reasoning layer of the Cascading Reasoning pyramid, are the logical-based formalisms on which OWL2 DL has been built. We introduce the syntax of a simplified DL, explaining the basic notions to understand the remainder of the paper. We refer the reader to Horrocks et al. [33] for a more thorough description of the OWL2 DL logic (SROIQ) and its semantics.

DL languages contain *concepts names* $A_{1},A_{2},\;\ldots$, *role names* P1,P2,… and *individual names* $a_{1},a_{2},\;\ldots$ A *role* *R* is either a role name $P_{i}$, its inverse $P_{i}^{-}$, or a complex role $R_{1}\circ \cdots \circ R_{n}$ consisting of a chain of roles. Concrete roles (or data properties) are roles with datatype literals (D) in the second argument. *Concepts* *C* are constructed from two special primitive concepts ⊥ (bottom) and ⊤ (top) or concepts names and roles using the following grammar:

$$C::=A_{i}\left|⊤|⊥|\neg C\right|C_{1}⊓C_{2}|C_{1}⊔C_{2}|\exists R_{1}.C_{1}|\forall R_{1}.C_{1}\left|\exists R_{1}.D_{1}\right|\forall R_{1}.D_{1}.$$

Note that the two last concepts are called, respectively, existential (∃) and universal (∀) quantifiers.

A Terminological Box (TBox) T is a finite set of concept (*C*) and role (*R*) inclusion axioms of the form

$$C_{1}⊑C_{2}\;\mathrm{and}\;R_{1}⊑R_{2}$$

with $C_{1}$, $C_{2}$ concepts and $R_{1}$, $R_{2}$ roles. A concept equation ($C_{1}\equiv C_{2}$) denotes that both $C_{1}$ and $C_{2}$ include each other:

$$C_{1}⊑C_{2}\;\mathrm{and}\;C_{2}⊑C_{1}.$$

An Assertion Box (ABox) A is a finite set of concept and role assertions of the form

C(a)andR(a,b)

with *C* a concept, *R* a role, and *a* and *b* individual names. We call the concepts assigned to an individual the types of the individual. A *Knowledge base* K=(T,A) combines T and A. I is an *interpretation* for K. I is a model of K if it satisfies all concept and role inclusions of T and all concept and role assertions of A. This can be written as I⊨K.

OWL2 contains three profiles, each limiting the expressivity power in a different way, to ensure efficiency of reasoning:

- OWL2 RL, which does not allow existential quantifiers on the right-hand side of the concept inclusion, eliminating the need to reason about individuals that are not explicitly present in the knowledge base. Furthermore, it does not allow quantified restriction, e.g., a minimum number of roles, a maximum number of roles or exactly a specific number of quantified roles. This profile is ideal to be executed on a rule-engine.
- OWL2 EL, which mainly provides support for conjunctions and existential quantifiers. This profile is ideal for reasoning over large TBoxes that do not contain, among others, universal quantifiers, quantified restrictions or inverse object properties.
- OWL2 QL, which does not allow, among others, existential quantifiers to a class expression or a data range on the left-hand side of the concept inclusion. This makes the profile ideal for query rewriting techniques.

Note that each of these profiles is a subset of OWL2 DL.

**Example** **2.** *In the ontology used to model our domain from our example in Section 1, we assign each Office various Policies. Based on these Policies, an Office can be considered a FlexibleOffice or not:*

$$\begin{array}{c} \mathrm{NoFixedHoursOffice}\equiv \mathrm{Office}⊓\exists \mathrm{hasPolicy}.\mathrm{FlexibleHours},\\ \mathrm{NoFixedHoursOffice}⊑\mathrm{FlexibleOffice},\\ \mathrm{StartEarlyOffice}\equiv \mathrm{Office}⊓\exists \mathrm{hasPolicy}.\mathrm{StartEarly},\\ \mathrm{StartEarlyOffice}⊑\mathrm{FlexibleOffice},\\ \mathrm{StopEarlyOffice}\equiv \mathrm{Office}⊓\exists \mathrm{hasPolicy}.\mathrm{StopEarly},\\ \mathrm{StopEarlyOffice}⊑\mathrm{FlexibleOffice}. \end{array}$$

*To model the observations that capture the various sensor readings across the city, we use the SSN Ontology [34]. We first model observations near flexible offices, and we then model observations near flexible offices that also capture congestion levels:*

$$\begin{array}{c} \mathrm{FlexibleOfficeObservation}\equiv \mathrm{Observation}\\ ⊓(\exists observedFeature.(\exists isLocationOf.\mathrm{FlexibleOffice}))\\ \mathrm{CongestionFOObservation}\equiv \mathrm{FlexibleOfficeObservation}\\ ⊓\exists observedProperty.\mathrm{CongestionLevel} \end{array}$$

*We can now model for each type of street, which is located near a flexible office, when it should be considered congested. With the congestion level defined as the number of detected vehicles divided by the street length (in meters):*

$$\begin{array}{c} \mathrm{HighTrafficMainRoadNearFlexibleOffice}\equiv \\ \;\mathrm{CongestionFOObservation}\\ \;⊓\exists observedProperty.\mathrm{MainRoad}\\ \;⊓\exists hasLocation.\mathrm{Location}\\ \;⊓\exists hasValue>0.025,\\ \mathrm{LowTrafficMainRoadNearFlexibleOffice}\equiv \\ \;\mathrm{CongestionFOObservation}\\ \;⊓\exists observedProperty.\mathrm{MainRoad}\\ \;⊓\exists hasLocation.\mathrm{Location}\\ \;⊓\exists hasValue<0.01. \end{array}$$

*Note that similar constructions can be made for different types of streets and that all these constructs are also subclasses of the concepts HighTrafficObservation or LowTrafficObservation.*

**Example** **3.** *In Example 2, we have modeled the TBox. Let us consider a minimal ABox A describing the office, the road, and their property:*

$$\begin{array}{c} Office(office),hasPolicy(office,pol1),\\ StopEarly(pol1),MainRoad(road),\\ CongestionLevel(prop),propertyOf(prop,road),\\ isLocationOf(road,office). \end{array}$$

*The observation capturing the current congestion level can be modeled as*

$$\begin{array}{c} Observation\left(obs_{i}\right),observedProperty\left(obs_{i},prop\right),\\ hasValue(obs_{i},0.03). \end{array}$$

*By applying reasoning, we can infer from K=(T,A) that*

$$\begin{array}{cc} K & ⊨FlexibleOffice(office),\\ K & ⊨FlexibleOfficeObservation(obs1),\\ K & ⊨CongestionFOObservation(obs1),\\ K & ⊨HighTrafficMainRoadNearFlexibleOffice(obs1),\\ K & ⊨HighTrafficObservation(obs1). \end{array}$$

### 3.2. Cascading Reasoning Generalization

Since Stuckenschmidt et al.’s vision of Cascading Reasoning was proposed, several new approaches populated the Stream Reasoning state of the art [17]. We slightly generalize the vision such that it is up to date with the latest developments within the Stream Reasoning domain.

The initial scope of reasoning frameworks was focused mainly on DL and DLP. Recently, temporal logics, non-monotonic LPs and technique for reasoning about time were proposed beside the traditional Stream Reasoning research areas. In the future, we also imagine the integration of on-line machine learning application, which already showed appealing results, and the combination of deductive and inductive reasoning [35,36].

In the original cascading reasoning pyramid, the role of RSP was limited to streaming data integration. Although this is utterly meaningful in combination with DL reasoning, data integration is a much more general problem to investigate when data are continuously changing. Moreover, RSP, but also stream processing, can support reasoning tasks (e.g., RSP under entailment or query rewriting).

**Figure 3:**
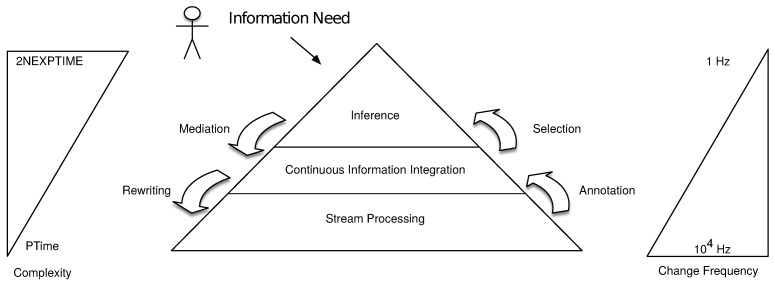
A generalization of Cascading Reasoning.

The updated and generalized cascading stream reasoning pyramid is depicted in Figure 3. As in the original vision, it aims at presenting the trade-off between expressiveness and rate of changes in the data.

We now detail each of the layers:

1. **Stream Processing:** At the lowest level, the data streams are processed. Different processing techniques can be used accordingly to the levels above, e.g., which information integration technique is used (if any). This layer can implement stream processing techniques like DSMSs and CEPs or use RSP when dealing with semantically annotated data. Moreover, this level can also solve part of the analytic needs, since it is able to compute descriptive analysis of the streaming data.
2. **Continuous Information Integration:** In order to achieve a high-level view on the streaming data, we need an information integration layer that offers a homogeneous view over the streams. The Continuous Information Integration layer combines data from heterogeneous streams into a common semantic space by the means of mapping assertions that populate a conceptual model. Two approaches are then possible to access the data: (i) Data *Annotation* (a.k.a. data materialization), i.e., data are transformed into a new format closer to the information need (ii) Query *Rewriting* (a.k.a. data visualization), i.e., the information need is rewritten into sub tasks that are closer to each of the original data formats.
3. **Inference:** In a cascading approach, an information need (IN) is formulated accordingly to a high-level view of the data. To enable efficient IN resolution, we need an inference layer that *mediates* the IN with domain-specific knowledge to the lower layers. Computational tasks at this level have a high complexity. This reduces the volume of data this level can actually process. Therefore, it is necessary to *select*, from the lower layers, the relevant parts of the streams that this layer has to interpret to infer hidden data. Possible inference implementations range from expressive reasoning, such as DL, ASP, metric temporal logic (MTL), or CEP, to machine learning techniques such as Bayesian Networks (BN) or hidden Markov models (HMM).

The original vision—which consists of raw stream processing, RSP, DL, and logic programming—fits this more general view: the raw stream processing is contained in our Stream Processing layer, RSP is contained in the continuous information integration layer, and DL & logic programming are part of the inference layer.

## 4. Cascading Reasoning with Streaming MASSIF

In this section, we explain how we realized Cascading Reasoning with Streaming MASSIF. We introduce the architecture of Streaming MASSIF and present a Domain Specific Language (DSL) that allows one to target the different layers of the cascading approach.

### 4.1. Layer Design

**Figure 4:**
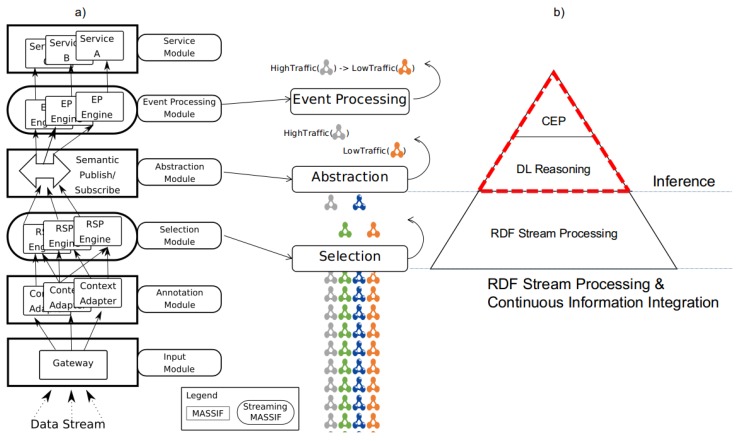
(**a**) Streaming MASSIF architecture and the alignment of the inference (consisting of the DL and CEP layer) and (**b**) stream processing layer with the event processing, abstraction, and selection modules.

In the following sections, we design a stream reasoning architecture that fulfills the *Objectives* and fits the generalized Cascading Reasoning vision. As depicted in Figure 4b, our approach consists of two layers that perform four tasks, starting from the bottom: (i) An RSP layer selects the parts of the streams that are relevant. (ii) It also integrates data from different streaming and static sources. (iii) An inference layer enriches the output of the previous layer by deriving implicit data using DL reasoning. (iv) It also performs temporal reasoning via CEP on the inferred abstractions.

### 4.2. Architecture

As discussed in Section 2, MASSIF is an event-driven platform that processes one event at a time and is, thus, not able to process streams nor capture temporal dependencies between events. However, its layered architecture and the ability to perform service composition over high-level concepts offer a good base to extend it into a Cascading Reasoning approach. We note that other platforms could have been used to realize our Cascading Reasoning approach; however, the layered architecture of MASSIF and enabled service subscription made it an ideal candidate.

To realize our cascading stream reasoning approach, two additional modules have been added on the MASSIF platform, as depicted by the rounded blocks in Figure 4a, i.e., a *Selection* and *Event Processing Module*. We named the resulting platform Streaming MASSIF. Compared to the original MASSIF platform, the *Selection Module* allows one to handle streaming data and select only the parts from the data stream that are relevant for further processing. These selections then can be abstracted in the *Abstraction Module*. The *Event Processing Module* allows one to detect temporal dependencies between events. Thus, the MASSIF platform allows services to subscribe to high-level events. Streaming MASSIF allows services to subscribe to data streams, extract high-level events, and detect temporal dependencies between those events. Furthermore, this can all be declaratively defined in a unifying language, which is further elaborated in Section 4.3.

#### 4.2.1. Selection Module

The **Selection Module** implements both the Stream Processing and the Continuous Information Integration Layer of the Cascading Reasoning approach and selects, through RSP, those parts of the RDF stream that are relevant. As depicted in Figure 4, the goal of this layer is to minimize that data stream and select only those parts of the stream that are relevant for further processing. We utilized YASPER [37], i.e., an RSP engine recently developed, that fully implements RSP-QL [31] semantics and can consumes RSP-QL queries. YASPER, differently from C-SPARQL [12] or CQELS [14] consumes time-annotated graphs instead of time-annotated triples. Only the selected data are forwarded to the next module. Note that multiple RSP engines can optionally run in parallel, for example, to distribute the load of various queries or handle multiple data streams.

**Figure 5:**
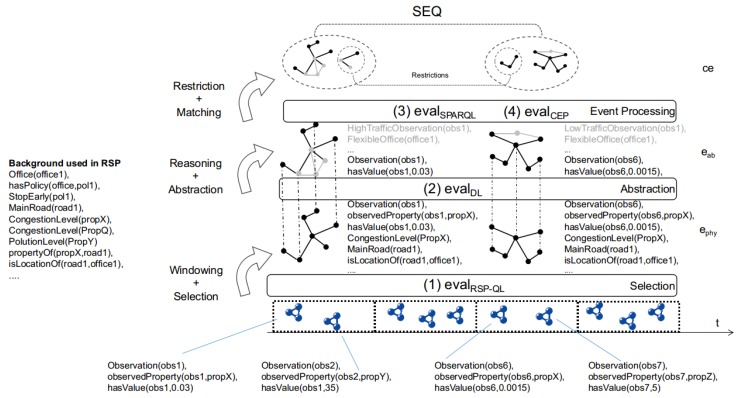
Processing steps of the Streaming MASSIF Cascading Reasoning Approach.

For example, at the bottom of Figure 5, a traffic observation data stream is visualized. As in a realistic situation, the events in the stream only describe that they are observations (e.g., Observation(obs1)), that they observe a certain property (e.g., observedProperty(obs1,propX)) and that a specific value has been observed (e.g., hasValue(obs1,0.03)). Note that these observations need to be combined with background knowledge to figure out if the event was observing congestion levels. Since we are only interested in traffic observations that can be considered high-traffic, we select only the congestion level observations in the stream with a value above 0.03 or below 0.01, as indicated in the domain knowledge. However, to determine that an observation is, in fact, a congestion level, we need to integrate with static background data describing the sensors. We also extract the information regarding the office near the location where the observation comes from, so we can determine later if these are flexible offices or not. Listing 1 shows a query *Q* that selects the relevant portion of the stream.

In Figure 5, this query will select Observation(obs1) and Observation(obs6) from the stream. It will also add some additional data to the event, such as information regarding the road and the offices that can be used in the next layer for the expressive reasoning step.

**Example** **4.** *(cont’d) One of the selected events describes the first observation in the stream:*

$$\begin{array}{c} Observation(obs1),\\ observedProperty(obs1,propX),\\ hasValue(obs1,0.03). \end{array}$$

*In the Selection Module, it has also been enriched with the following data:*

$$\begin{array}{c} CongestionLevel(propX),\\ Office(office1),\\ MainRoad(road1),\\ isLocationOf(road1,office1),\\ StopEarly(pol1),\\ hasPolicy(office1,pol1). \end{array}$$

**Listing 1:** Example of the RSP-QL Query used in the Selection Module.

          CONSTRUCT {
                  ?obs_X a ssn:Observation.
                  ?obs_X ssn:observedBy ?sensor_X.
                  ?obs_X ssn:observedProperty ?property_X.
                  ?property_X a CongestionLevel. 
                  ?obs_X hasValue ?value. 
                  ?property_X isPropertyOf ?foi. 
                  ?foi isLocationOf ?loc. 
                  ?loc hasPolicy ?pol. } 
          FROM NAMED WINDOW :traffic [RANGE 5m, SLIDE 1m] ON STREAM :Traffic
          WHERE {
                  ?property_X a CongestionLevel.
                  ?property_X isPropertyOf ?foi.
                  ?foi isLocationOf ?loc.
                  ?loc hasPolicy ?pol.
                  WINDOW ?w {
                          ?obs_X a ssn:Observation.
                          ?obs_X ssn:observedBy ?sensor_X.
                          ?obs_X ssn:observedProperty ?property_X.
                          ?obs_X hasValue ?value.
                          FILTER(?value > 0.03 || ?value < 0.01)
                   }
          }
          


#### 4.2.2. The Abstraction Module

The **Abstraction Module** implements the DL inference sub-layer. It receives the selected events from the *Selection Module* and abstracts them to high-level concepts. The *Abstraction Module* consists of a semantic publish/subscribe mechanism and allows the subscription to abstracted events, through high-level concepts. Each service in the service module can subscribe to events by defining event descriptions.

Technically, the *Abstraction Module* operates on an OWL reasoner, i.e., the HermiT [15] reasoner (note that, due to the modularity of the platform, other reasoners can easily be plugged in). Each time events have been selected in the *Selection Module*, they are added to the ontology in the *Abstraction Module*. Through the use of reasoning, we check which inferred types of the individuals are the types that one of the services subscribed to. When these types are found, the abstracted events are constructed using the found types, the underlying event, and the processing time. The abstracted event is then forwarded to those services that subscribed to the found types. Lastly, the events are removed from the ontology ABox. When new events have been selected by the underlying module, they are added to the ontology and the types of new events can be checked.

**Example** **5.** *(cont’d) The selected events from the Selection Module can now be abstracted according to the defined ontology in Example 2. Through reasoning we obtain that*

$$\begin{array}{c} HighTrafficStreet(obs1),\\ HighTrafficMainRoadNearFlexibleOffice(obs1),\\ HighTrafficObservation(obs1),\\ FlexibleOffice(office1). \end{array}$$

*Let us assume that a service is interested in all HighTrafficObservations. The selected event, enriched with the inferred types, is forwarded to that service or to its Event Processing Module.*

#### 4.2.3. The Event Processing Module

The **Event Processing Module** implements the temporal reasoning sub-layer. When event processing is necessary, the *Event Processing Module* receives the abstracted events from the *Abstraction Module*. Each of the received abstracted events is checked if it matches an event pattern, through the use of the Esper CEP engine (http://www.espertech.com/esper/). We choose Esper since it supports the declarative language EPL. Note that, when multiple abstracted events are inserted at once, they are first ordered according to their timestamp. We allow one to define additional filter restrictions, such that the patterns can be matched on a fine-grained level.

In CEP, *filter restrictions* can be defined on the event values, e.g., Event A (speed = 45) has the property *speed* with a value of 45, and one can restrict events to have speed values above a certain threshold. *Join restrictions* can be defined over events, e.g., if each event type has a location A (location = loc1) and B (location = loc1), then we can impose the restriction that Events A and B should have the same location. We allow one to define additional queries to specify both restrictions.

**Example** **6.** *(cont’d) Let us assume that the pattern defined in the Event Processing Module is looking for all HighTrafficObservations followed by LowTrafficObservation within 10 min, which detects decreasing traffic. This can be defined through the pattern:*

EVERY HighTrafficObservation SEQ LowTrafficObservation WITHIN10m.

*We need to add additional restrictions to ensure that both the HighTrafficObservation and low TrafficObservation occurred in the same street. This can be done by filtering on the location. We know from Example 2 that each HighTrafficObservation should have a hasLocation relation. Therefore, we can enforce that they should be linked to the same location. In Section 4.3, we show how this can easily be defined.*

When filter restrictions have been defined, these restrictions are checked first before adding the event to the CEP engine. When a join-restriction has been detected (e.g., the *TrafficObservations* should have the same location), the bindings of those variables are used within the CEP engine to perform the joins. When an event pattern matches, it is forwarded to the associated service.

#### 4.2.4. The Remaining Modules

The MASSIF platform also consists of an **Input Module** that serves as the entry point of the platform and an **Annotation Module**, where raw data can be semantically annotated if necessary.

Finally, the **Service Module** receives the processed data and can perform additional analysis. Through the **Service Module**, information needs formulated using our DSL (see Section 4.3) can be issued to Streaming Massif. Therefore, services can subscribe to all underlying modules with one query.

**Listing 2:** Syntax of the Streaming MASSIF DSL.

            DSL -> NameSpace* EventDecl* RSPQL?
            EventDecl -> ‘NAMED EVENT’ EventName (AbstractEvent | ComplexEvent)
            AbstractEvent -> ‘AS’ DLDescription
            ComplexEvent -> ‘MATCH’ (Modifier)? EventPattern (Guard)? (IFClause)?
            EventPattern -> EventPattern EventOperator EventPattern|AbstractEvent|‘NOT’EventPattern
            IFClause -> ‘IF’ ‘{’ (‘EVENT’ AbstractEvent ‘{’ BGP ‘}’)* ‘}’
            EventOperator -> ‘AND’ | ‘OR’| ‘SEQ’
            Modifier -> ‘EVERY’ | ‘FIRST’ | ‘LAST’
            Guard -> ‘WHITIN’ Num ‘(’ TIMEUNIT ‘)’
            TIMEUNIT -> ‘s’ | ‘m’ | ‘h’ | ‘d’
            EventName -> String
            Num -> [0-9]+
            NameSpace -> SPARQL PREFIX SYNTAX
            DLDescription -> MANCHESTER SYNTAX
            BGP -> SPARQL BGP SYNTAX
            RSPQL -> RSP-QL SYNTAX
          


### 4.3. A Domain Specific Language for Streaming MASSIF

In this section, we introduce a DSL that allows users to formulate information needs by using the proposed Cascading Reasoning approach. In order to explain the DSL, we provide an example of information need and we explain how each part of the query maps to the different module described in Section 4.2.

Listing 2 describes the grammar of the proposed query language. Note that for conciseness reasons, we did not incorporate the following sub-grammars:

1. DLDescription: The definition of the abstract event types is based on the Manchester syntax. For more information regarding this syntax, we refer the reader to the Manchester W3C page (https://www.w3.org/TR/owl2-manchester-syntax/).
2. BGP: In the definition of the complex events, one can define Basic Graph Pattern (BGP) for restricting the validity of the events. We did not incorporate the explanation of the syntax of BGP in this proposal.
3. RSPQL: For targeting the RSP module, we utilize RSP-QL. The full syntax of RSP-QL has not been incorporated in our syntax proposal, more information regarding RSP-QL can be found in Dell’Aglio et al. [31].

**Figure 6:**
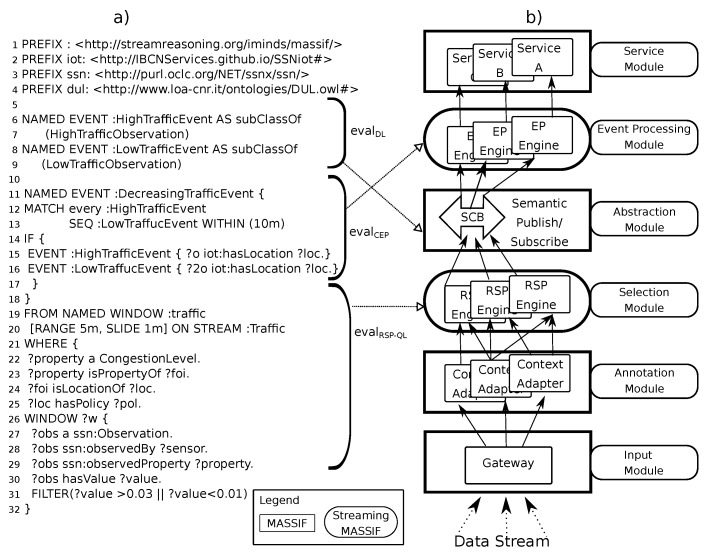
(**a**) Example of the Streaming MASSIF DSL and how it targets the Streaming MASSIF achitecture (**b**).

As defined in Listing 2, an information need comprises multiple namespaces (*NameSpace*), multiple event declarations (*EventDecl*) and an optional *RSPQL* declaration. Figure 6a shows an information need from the example use-case. We now explain how this DSL targets each module of the cascading stream reasoner.

#### 4.3.1. DSL Fragment for the RSP Layer

From Line 19 in Figure 6a, the RSP-QL syntax is used for selecting the relevant events from various streams. Note that there is no query form defined, since we restrict the use to the construct query form. The construct query template is generated from the BGP in the WHERE clause. This part of the query targets the *Selection Module* of the Streaming MASSIF architecture. Note that the definition of the RSP-QL clause is optional in the language. In the absence of the RSP-QL clause, all streaming data is directly processed by the next layer (i.e., the abstraction layer). In this case, each event in the stream is processed one by one.

#### 4.3.2. DSL Fragment for the DL Sub-Layer

An information need typically requires one to define multiple events. An event declaration (*EventDecl*) starts with the declaration of a *NAMED EVENT*, a name for the event (*EventName*), and either the definition of an abstract event (*AbstractEvent*) or a complex event (*ComplexEvent*). The abstract event definition start with the *‘AS’* keyword to indicate how the event name should be interpreted, followed by a declaration in Manchester DL syntax. This is shown in Figure 6a on Lines 6–9. We chose the Manchester Syntax (https://www.w3.org/TR/owl2-manchester-syntax/) for the definition of these events since its very concise and expressive.

The defined abstracted event definitions are used in the *Abstraction Module* to indicate the high-level concepts that should be abstracted and forwarded to the next layers.

#### 4.3.3. DSL Fragment for the CEP Sub-Layer

Besides the *AbstractEvents*, the *EventDecl* clause can also define complex events (*ComplexEvents*). These are declared with the ‘MATCH’ keyword, followed by a modifier (*Modifier*), an event pattern (*EventPattern*), a guard (*Guard*), and an optional restriction clause (*IFClause*). The *EventPattern* is constructed from various abstract events and event operators (*EventOperators*). These declarations are used within the *Event Processing Module*. Figure 6a shows an example event pattern defined over high and low traffic abstractions on Lines 11–13.

The restrictions (*IFClause*) are declared using the ‘IF’ keyword, followed by the abstract event name used in the pattern that needs to be restricted. The restriction itself is defined in a BGP. Both filter and join restrictions can be modeled in this manner. An example on how to define join restrictions over multiple events can be found in Figure 6a on Lines 14–16. The restriction states that the high and low traffic abstractions should occur in the same location. Note that the variable name ‘loc’ is the same in both restrictions.

We can also define restrictions to filter individual events. Listing 3 shows a filter restriction example over the high and low traffic abstractions that restricts observations to be after 3 o’clock in the afternoon.

**Listing 3:** DSL Event Restriction Clause Example.

          1          NAMED EVENT :DecreasingTrafficEvent {
          2          MATCH EVERY :HighTrafficEvent
          3          SEQ :LowTrafficEvent WITHIN (10 m)
          4          IF {
          5                 EVENT :HighTrafficEvent { ?o timeStamp ?time.
          6                  FILTER(hours(?time) > 15) }
          7                 EVENT :LowTrafficEvent { ?o2 timeStamp ?time2.
          8                  FILTER(hours(?time)>15) } }
          9          }
          


Note that the SPARQL FILTER clause is optional. When the defined BGP does not match the underlying event, i.e., no results are returned, the event is filtered out and not considered in the complex event processing.

## 5. Streaming MASSIF’s Formalization

Now that we have described the architecture we formalize how the different layers of Streaming MASSIF collaborate. We do this by focusing on the Cascading Reasoning pyramid that abstracts Streaming MASSIF, as shown in Figure 4b. The cascading approach consists of CEP and DL as inference methods and RSP for continuous information integration.

### 5.1. RDF Stream Processing Layer

The RSP layer receives RDF streams (as defined in Definition 1) as input and answers continuous queries written in RSP-QL (see Definition 6). A given RSP-QL query *Q* is evaluated against a RSP-QL dataset *SDS* (as defined in Definition 5). The result of the defined queries is forwarded to the next layer. Therefore, we fix the Query Form to the CONSTRUCT query form.

### 5.2. Continuous Information Integration Layer

As we previously mentioned, we assume that data streams arrive directly encoded as RDF streams. This assumption allows us to perform stream processing and continuous information integration in the RSP layer by means of a common vocabulary.

Notably, we do not consider the annotation task (a.k.a. the data materialization task) as part of the approach. If the data are not natively RDF streams, approaches such as TripleWave [38], which rely on mapping techniques such as RML [39] and R2RML (https://www.w3.org/TR/r2rml/), can be utilized. Note that the *Annotation Module* in the Streaming MASSIF architecture can be used for this goal.

### 5.3. The Inference Layer

The inference layer of our architecture consists of two sub-layers: (i) *Description Logics*, since we want to infer information not explicitly available in the streams, and (ii) *Temporal Logics*, because we aim at deducing information based on temporal relations between the data.

In the following, we explain how we link those sub-layers together.

First, we need to make a distinction between physical events and abstract events:

**Definition** **7.** *A* ***physical event*** *$e_{phy}$ is an event that occurs directly in the input stream S or is a result of the RSP layer. Note that, in the latter, the event may also include background data. A collections of physical events is defined as $E_{phy}$.*

In Figure 5, multiple physical events are depicted in a stream. Four physical events are detailed. Example 4 describes the physical event that contains the first observation in the stream. Note that, in the RSP layer, the physical events can still be enriched with additional information.

**Definition** **8.** *A* ***subscription TBox*E** *consists of those TBox concepts that have been used as high-level concepts in a service subscription. It bridges the gap between domain ontology and the physical events. E contains all the NAMED EVENTS defined for the Abstraction Module.*

The use of E allows us to select only those physical events that services are actually interested in. From these physical events we derive abstract events:

**Definition** **9.** *An**abstract event**$e_{ab}$ consists of one or more physical events $e_{phy}$ and hides their low-level details. An abstracted event $e_{ab}$ can be inferred under an entailment* Σ *from a collection of physical events $E_{phy}$ iff $\exists e_{i}\in E_{phy}:\left(T^{+},A^{+}\right)⊨C_{E}\left(e_{i}\right)$ with $C_{E}\in E$ and $K=(T^{+},A^{+})$, with K the knowledge based used in the reasoning process. $T^{+}=T\cup E$ is the TBox and $A^{+}$ the ABox, with $A^{+}=A\cup E_{phy}$. We can now define the abstracted event as the triple $e_{ab}=\left(C_{E},E_{phy}^{\prime },t\right)$, with $E_{phy}^{\prime }$ the collections of physical events in $E_{phy}$ that lead to infer $C_{E}\left(e_{i}\right)$ and t the processing time at which the first physical event in $E_{phy}^{\prime }$ was produced. $E_{ab}$ represents a collection of abstracted events. This is the case when multiple abstracted events can be abstracted.*

**Example** **7.** *(cont’d) In the DSL defined in Figure 6a, the services subscribed to HighTrafficObservations by defining the named event HighTrafficEvent. Let us assume that only HighTrafficEvent is contained in E. The physical events can now be abstracted according to the defined ontology in Example 2. Through reasoning we obtain that*

$$\begin{array}{c} HighTrafficObservation(obs1),\\ HighTrafficMainRoadNearFlexibleOffice(obs1),\\ FlexibleOffice(office1),\\ HighTrafficEvent(obs1). \end{array}$$

*This results only in the abstracted event $\left(HighTrafficEvent,e_{phy},t_{i}\right)$ with $e_{phy}$, the physical event, and $t_{i}$, the time $e_{phy}$ is produced, since only HighTrafficEvent is defined in E.*

We now want to identify temporal dependencies between the abstracted events provided by the DL sub-layer.

We build upon the definitions from CEP to detect the temporal dependencies between abstracted events provided by the DL sub-layer.

**Definition** **10.** *An****event pattern*EP***is a statement of the form*

$$\left[▿\right]\left(E_{1}\wedge \cdots \wedge E_{k}\right)|\left(E_{1}\vee \cdots \vee E_{k}\right)\left[▵\right]$$

*with $E_{i}$ either (i) an event type, (ii) a complex event using AND, OR, NOT, or SEQ, or (iii) another event pattern (recursively).* ∇ *is an optional modifier, e.g., EVERY, and* ∆ *is an optional guard, e.g., WITHIN.*

We use these patterns to instantiate complex events that represent inferred information.

**Definition** **11.** *A* ***complex event*ce** *definition is a triple ce=(h,p,R) with*

- *h as the complex event type,*
- *p as the pattern defined using operators, modifiers, and guards, and*
- *R as a set of restrictions.*

*h is instantiated when p and R are satisfied.*

The set of abstracted events (i.e., the collection of triples $\left(C_{E},e_{phy},t\right)$) is used in the event pattern matching. More specifically, each type $C_{E}$ is checked if it matches the event types within the pattern. Additionally, the *restrictions* $R=(CR_{E},q_{SPARQL})$ can be defined on each event type in an event pattern. $CR_{E}$ is an event type (i.e., defined in E) and $q_{SPARQL}$ is a SPARQL query. The SPARQL query is evaluated over each $e_{phy}$ contained in the abstracted event ($e_{ab}=\left(C_{E},e_{phy},t\right)$), where $C_{E}==CR_{E}$. Restrictions over multiple events in the event pattern can be achieved by creating multiple restrictions *R* with the same variable names in the $q_{SPARQL}$. The variable bindings are extracted and used for joining the events. This is shown in the restrictions of Example 8 through the use of the reoccurring variable name “?loc”.

**Example** **8.** *(cont’d) To detect the decreasing traffic, we need to monitor for a high amount of traffic near flexible offices followed by low amounts of traffic near the same flexible offices within a certain time range. This can be done by defining the complex event definition triple: $ce=(CE_{E},p,R)$ with*

- *$CE_{E}$ as the complex event type DecreasingTraffic,*
- *p as the pattern describing EVERY HighTraffic- AbstractionSEQLowTrafficAbstraction*
*WITHIN10m, and*
- *R as a set of restrictions of the form $\left(CR_{E},q_{SPARQL}\right)$ consisting of*
* (HighTrafficAbstraction, $q_{1})$ with $q_{1}=$
    1    Select * WHERE {
    2    *?o ssniot:hasLocation ?loc.}*
* (HighTrafficAbstraction, $q_{2})$ with $q_{2}=$
    1    Select * WHERE {
    2    *?o2 ssniot:hasLocation ?loc.}*

*Note that the restrictions state that high and low traffic events need to have the same location. The value in ?loc will be used to restricts the complex events, since its the only variable with the same name in $q_{1}$ and $q_{2}$.*

### 5.4. Unified Evaluation Functions

In the following, we explain, by means of Figure 5, how to combine the different layers into a single evaluation framework. At the lowest level, we have the evaluation of the RSP layer. Let us consider an RSP-QL query *Q*. The evaluation of *Q* over dataset *SDS* is defined as

Ω(t)=eval(SDS,SE,t),witht∈ET

where ET represents all the time instances where SDS is defined, and Ω is a time-varying multiset of solution mappings that maps time *T* to the set of solution mappings multisets [31]:

Ω:T→{ω|ωisamultisetofsolutionmappings}.

We consider only the CONSTRUCTS query form; therefore, the solution mappings still need to be substituted in a graph template defined in the query (as defined in the SPARQL 1.1. specification: https://www.w3.org/TR/sparql11-query/construct):

$$G_{\Omega }\left(t\right)=\sigma \left(G_{template},\Omega \left(t\right)\right).$$

with σ the substitution function and $G_{template}$ the graph template defined in *Q*. The solution $G_{\Omega }\left(t\right)$, for each t∈ET, is a subset of the data in *SDS* and is sent to the next layer in the cascading reasoner for further processing. We can define the evaluation of the RSP layer as

$$eval_{RSP-QL}\left(SDS,Q\right)=G_{\Omega }\left(t\right),\forall t\in ET.$$

Each time the RSP layer produces results, they are sent to the DL layer as a set of physical events E${}_{phy}=G_{\Omega }\left(t\right)$. The DL layer converts the physical events $E_{phy}$ to a set of abstracted events $E_{ab}$ under a certain entailment Σ.

$E_{ab}=\left\{C_{E}\left(e_{i}\right)\right|\exists e_{i}\in E_{phy}:\left(T^{+},A^{+}\right)⊨C_{E}\left(e_{i}\right)\wedge C_{E}\in E$ with $T^{+}=T\cup E$ and $A^{+}=A\cup E_{phy}\}$. The $eval_{DL}$ reasoning step is defined as

$$eval_{DL}\left(E_{phy},E,O,\Sigma \right)=E_{ab}$$

where $E_{ab}$ is the set of abstracted events and the quadruple $<E_{phy},E,O,\Sigma >$ comprises the following:

- $E_{phy}$—a set of one or more selected physical events contained in $G_{\Omega }\left(t\right)$.
- O—the ontology describing the domain knowledge. O=(T,A) with T the TBox and A the ABox describing O.
- E—an ontology TBox that bridges the domain ontology O and the physical events $E_{phy}$. This describes formally the abstraction based on O. Only the concepts in E will be considered as abstracted events.
- Σ—the entailment regime under which the reasoner has to extract the abstract events from $E_{phy}$.

Finally, we define the result of the evaluation of the CEP layer as a set of abstract events:

$$eval_{CEP+}\left(CE,E_{ab}\right)=\left\{\left(CE_{E},⋃e_{phy},t\right)\right\}$$

with $CE_{E}$ the complex event type of the complex event ce∈CE that matched, $e_{phy}$ the physical events in $E_{ab}$ that cause the patterns to trigger and *t* the processing time at which the patterned triggered. In the resulting complex event, the union of the underlying physical events is taken and the complex event type is assigned.

Since complex events are still physically represented as RDF graphs, in order to evaluate restrictions we can simply extend $eval_{CEP}$ with $eval_{SPARQL}$ that evaluates the restrictions describes as SPARQL queries.

To ensure termination, we restrict to non-recursive pattern definitions, i.e., $\forall p\in CE,∄E\in p:CE_{E}==E$. The complex event type is thus not allowed in the definition of the pattern.

### 5.5. Summary

To conclude, we described a stream reasoning stack that is able to (a) select the relevant portions of the stream using RSP, (b) abstract the selected RDF graphs using expressive reasoning techniques and selecting only those that match the expected abstractions, and (c) apply complex event processing over these abstractions to detect temporal dependencies.

## 6. Evaluation

To evaluate Streaming MASSIF, we extended the City Bench benchmark [4] with expressive ontology concepts, as those described in Example 2. We also extended the ABox and added various offices located near the monitored streets, each with a set of random policies. Among these office policies is the possibility to start early, to stop early, and to have flexible work hours and the presence of childcare. To further increase the complexity, we also added some complex roles which are used within the high and low traffic modeling, e.g.,

$$\begin{array}{c} observedFeature⊑observedProperty\;\circ isPropertyOf \end{array}.$$

For streaming the City Bench data, we utilized RSP Lab (https://github.com/streamreasoning/rsplab) and ran the streamers on a different node. The evaluation was conducted on a 16 core Intel Xeon E5520 @ 2.27 GHz CPU with 12 GB of RAM running on Ubuntu 16.04.

We first show the need for Cascading Reasoning when dealing with high-volatile streams.

### 6.1. The Need for Cascading Reasoning

To illustrate the need for Cascading Reasoning, we first show that current approaches have problems performing expressive reasoning over high-volatile streams. For now, we do not consider the temporal aspect. Reasoning techniques exist with different trade-offs between expressivity of reasoning and complexity of processing. Very low expressive reasoners are more performant as their complexity of processing is lower. We compare various reasoners within the spectrum of expressivity.

#### 6.1.1. Setup

To show the need for Cascading Reasoning, we provide batches of events, ranging between different number of events, to various reasoning techniques and measured the time it took each engine to process a specific number of events. The events themselves were captured from a City Bench event stream and the extended City Bench ontology was utilized to perform the reasoning. As the expressivity of each reasoning approach differs, we calculated the correctness of each engine. The correctness is measured as the percentage of concepts in the ontology that can be correctly calculated considering the expressivity of the reasoner.

The throughput is calculated by serving batches of 1, 10, 100, 1000, and 10,000 traffic observation events and calculating how long each approach takes, on average, to process the events. The batches are considered, as data is typically windowed when considering streaming data.

#### 6.1.2. Results

**Figure 7:**
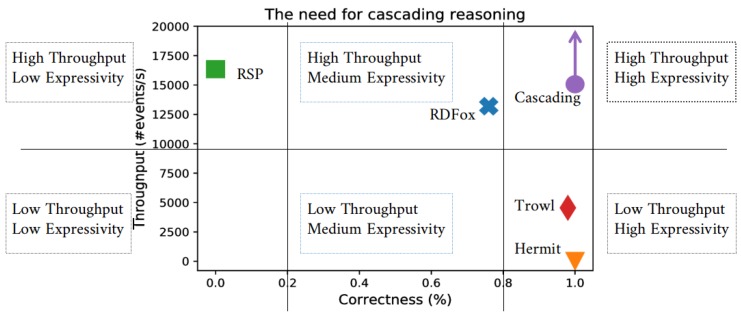
The comparison in terms of throughput and correctness for various approaches. A monolithic approach needs to trade off correctness for performance, whereas combined Cascading Reasoning approaches can cover both. This allows one to achieve high throughput and high expressivity.

Figure 7 shows a comparison of various reasoners in terms of throughput and correctness, while Table 1 provides the processing time for each reasoner in function of the number of events processed in each batch. HermiT was not able to handle batch sizes larger than 10,000 events due to out-of-memory exception. Therefore, the averages in Figure 7 are taken over batch sizes between 1 and 10,000 events.

RSP engines typically have very low to no reasoning capabilities, as they specifically aim at processing high volatile streams. As depicted in Figure 7, they have a high throughput, but very low expressivity as their correctness is very low. RDFox [16] is the fastest reasoner currently available. It supports OWL2 RL reasoning, a subset of OWL2 DL reasoning. It does not consider various ontology construction in order to achieve high performance. As can be seen in Figure 7, its throughput is rather high but it is not completely correct, as it lacks the expressivity to reason about all concepts correctly. Hermit [15] is a fully fledged OWL2 DL reasoner consisting of the needed expressivity to reason correctly about OWL2 DL ontologies. However, due to this expressivity, it is rather slow. TrOWL [40] is an OWL2 DL reasoner that allows one to perform approximation to enable stream reasoning over ontologies. Its throughput is higher than HermiT but lower than RDFox. However, its expressivity is higher than RDFox’s but lower than HermiT’s, as it does not support all OWL2 DL concepts. We show that, by combining the highest throughput approach, i.e., RSP, with the highest expressivity approach, i.e., HermiT, we can achieve both a high throughput and high expressivity approach. This is depicted as *Cascading* in Figure 7. The processing time is not simply the addition of the two layers. The speedup is achieved because of two reasons. The first reason is that the RSP layer can select only relevant parts of the streams to be processed with the expressive reasoner, resulting in fewer events being processed in the second layer. The second reason is that a lower amount of background data is necessary in the second layer, as the tasks of integrating the sensor data with the background data can now be performed in the RSP layer. From there, the relevant information for further processing can be selected and used in the second layer. The arrow indicates that possible higher throughputs can be achieved by duplicating and distributing the various parts of the Cascading Reasoning approach. For example, multiple streams can first be processed with its dedicated RSP engine before the results are combined and processed with a higher expressivity approach. This scalability is not possible with the other approaches, as they are monolithic systems. We also note that 78% of the IoT-labeled ontologies in the Linked Open Vocabularies repository (lov.linkeddata.es) (we only considered the ontologies that were accessible at the time of writing) require the OWL2 DL expressivity to infer all concepts correctly.

### 6.2. Test 1: Increasing Event Rate

**Figure 8:**
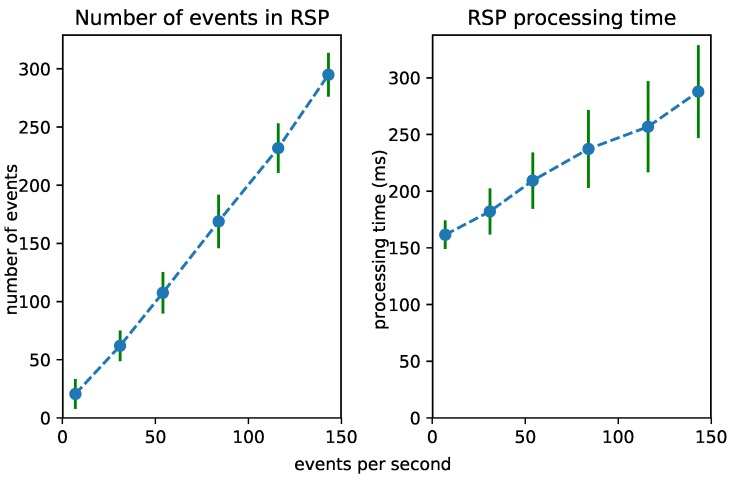
The influence of an increasing event rate on the number of the to-be-processed events (**left**) and the performance (**right**) of the Selection Module.

**Figure 9:**
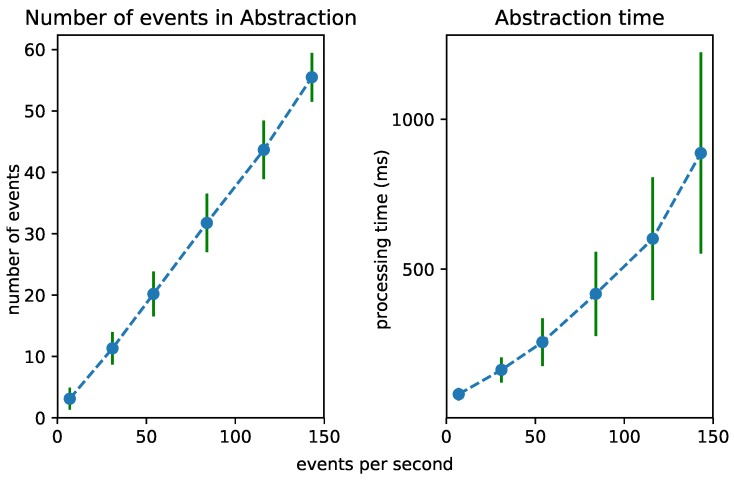
The influence of increasing event rate on the number of the to-be-processed events (**left**) and the performance (**right**) of the Abstraction Module.

**Figure 10:**
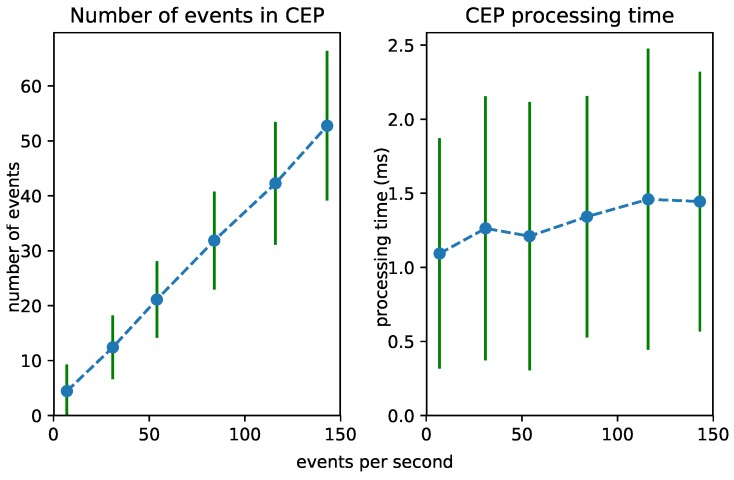
The influence of increasing event rate on the number of the to-be-processed events (**left**) and the performance (**right**) of the Event Processing Module.

To test the scalability of the Streaming MASSIF itself, we first artificially sped up the traffic streams to see how many events the platform can handle. Each stream in City Bench produces data every 5 min. We sped up the stream to produce multiple events per second. Figure 8, Figure 9 and Figure 10 visualize for each component the number of processed events and the processing time for a specific event rate. The RSP processing time in Figure 8 denotes the time taken to select the events within the window, the Abstraction time in Figure 9 denotes the time taken to abstract the received events from the RSP layer to high-level concepts, and the CEP processing time in Figure 10 measures how long it takes for the pattern to match, when the last event that causes the pattern to match arrives. On the *x*-axis, for all of the figures, we plotted the (rounded) actual event rate as they entered the platform. Note that, each time the stream produces data, five observations are produced: the average speed, the vehicle count, the measured time, the estimated time, and the congestion level. However, it is not stated explicitly in the stream what kind of observation is transmitted. Integrating with background knowledge is thus required to filter out the congestion level observations. This is performed in the RSP layer. We evaluated our results over eight streams and calculated the averages over the first 120,000 events. To easily calculate the processing time in each layer, we used a tumbling window (the sliding parameters is the same as the window width) of 2 s for each event rate. Using a tumbling window, each event only occurs once, and this simplifies the processing time calculations. To perform the evaluation, we used the example query from Figure 6a.

From Figure 8, we can see that the greatest selection of events happens in the RSP layer, while fewer events are selected in the abstraction layer. This is clear, since the number of events in the abstraction decreases when forwarded to the *Event Processing Module*, as depicted in Figure 10. Furthermore, the processing time in the Abstraction layer rises more quickly than it does in the other layers, which can be expected of an expressive reasoning process. However, we see that, when abstracting even more than 50 events, the abstraction time is lower than 1 s. The total latency of the abstraction remains well below 2 s (the size of the window), and the system thus stays reactive even when processing 300 events per second.

**Figure 11:**
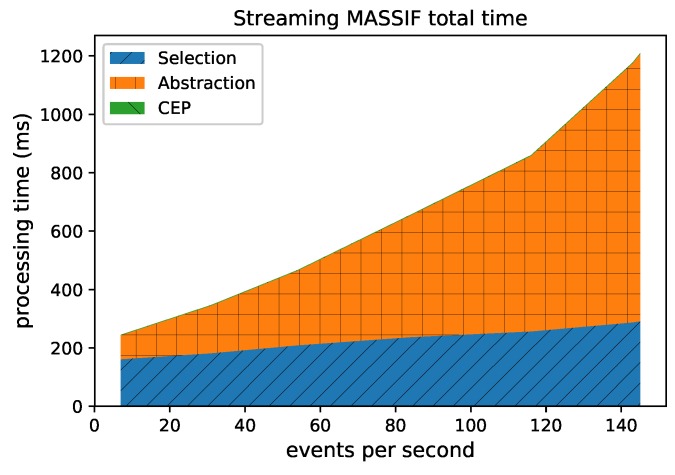
The influence of increasing event rate on total processing time of Streaming MASSIF and its components.

Figure 11 shows the influence of the event rate on the different layers combined. It is clear that the abstraction is most influenced by the event rate. This is because more events need to be abstracted. As the time for the event processing is very low, it is hardly visible in the graph.

### 6.3. Test 2: Increasing Window Size

**Figure 12:**
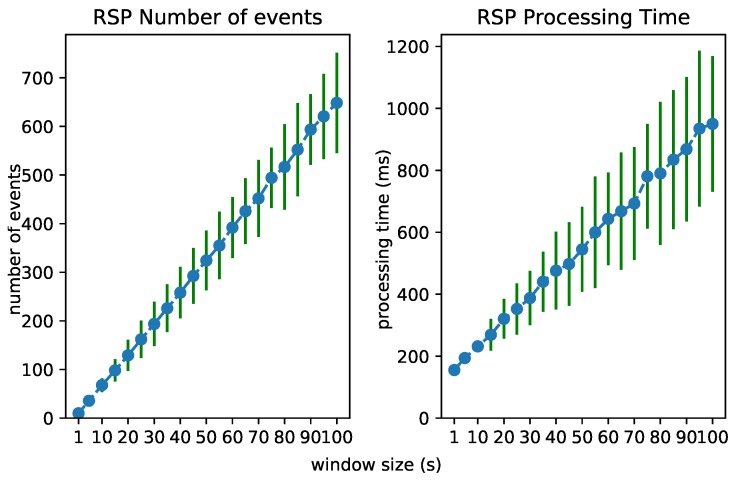
The influence of increasing the window size on the number of the to-be-processed events (**left**) and the performance (**right**) of the Selection Module.

**Figure 13:**
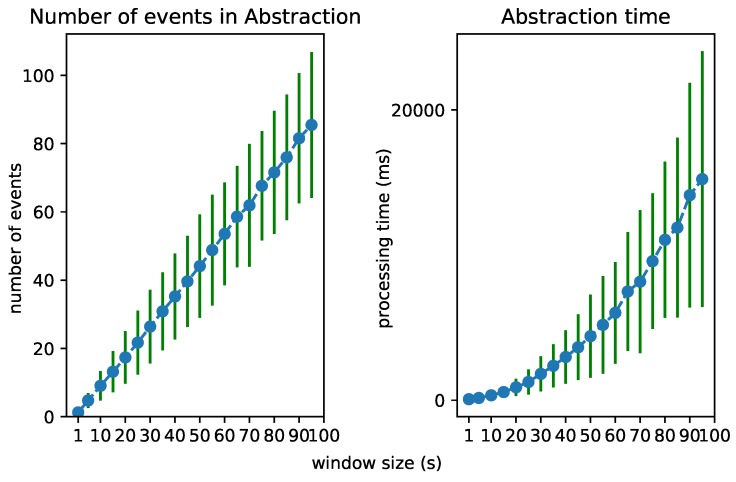
The influence of increasing the window size on the number of the to-be-processed events (**left**) and the performance (**right**) of the Abstraction Module.

**Figure 14:**
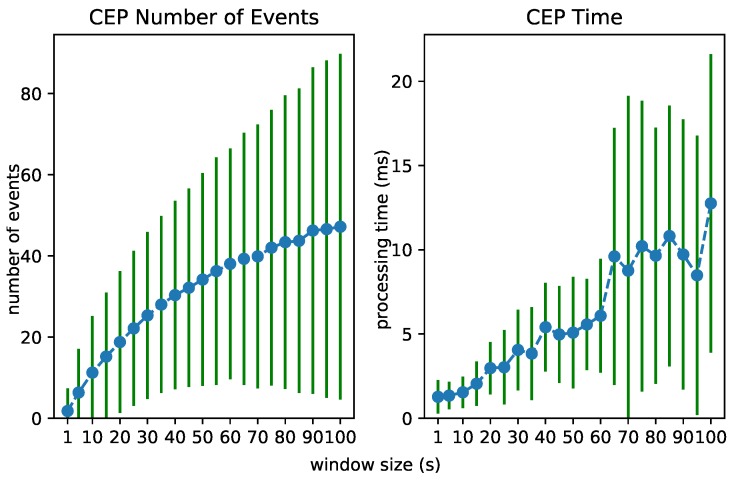
The influence of increasing the window size on the number of the to-be-processed events (**left**) and the performance (**right**) of the Event Processing Module.

The performance of each layer is clearly dependent on the number of considered events. We investigated the processing time of each layer when the window size in the RSP layer increases. This forces the processing of an increasing number of events in each layer. Figure 12, Figure 13 and Figure 14 visualize the number of processed events and the processing time for each layer when the window size increases from 1 to 100 s. We see a clear increase in the processing time of each layer. The Abstraction time increases exponentially, which can be expected of an expressive reasoning process. However, abstracting up to 100 events takes about 15 s, still much faster than the 100 s it takes for the window to slide.

### 6.4. Test 3: The Influence of the Selection Rate

**Figure 15:**
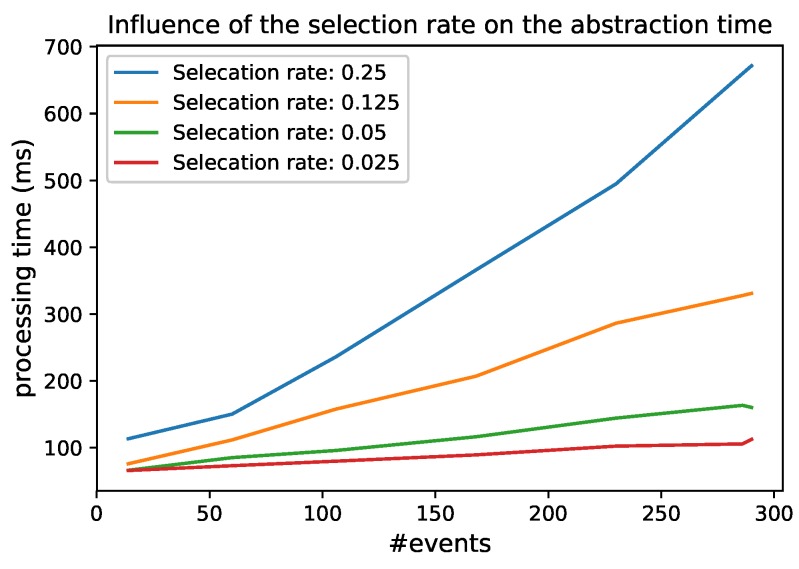
The influence of the selection rate on the abstraction time.

Besides the size of the window, the percentage of events that are selected in the *Selection Module* influences the abstraction time in the *Abstraction Module*. This is because the more events that are selected, the more events that need to be abstracted through expressive reasoning, and the expressive reasoning is expensive. Figure 15 shows the influence of a decreasing selection rate on the abstraction time. When the selection rate decrease, the number of events that need to be abstracted decreases and has thus less influence on the reasoning time. It is thus important that the *Selection Module* carefully selects only the relevant events.

### 6.5. Test 4: Comparison with MASSIF

**Figure 16:**
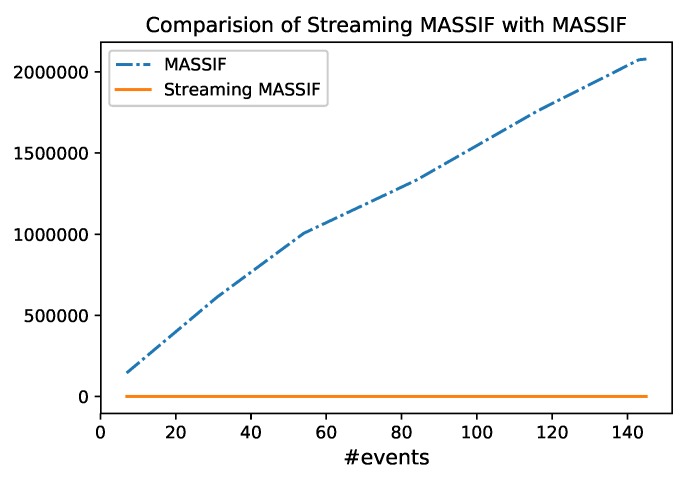
Throughput of Streaming MASSIF and MASSIF.

Since we extended the MASSIF platform to implement the adapted Cascading Reasoning vision, we also measure how fast the MASSIF platform could process the event stream. Note, however, that the MASSIF platform needs to perform the abstraction on all the background data, consisting of all the information of all the sensors, the streets, the offices, etc. All of the background data contain more than 60,000 statements. In the RSP layer, we select the relevant portion from the stream but also select the relevant data from the background knowledge. This eliminates the need for the Abstraction layer to contain the whole background knowledge. The TBox is most important there. Without this selection step, the abstraction of a single event in the MASSIF platform takes up to 20 s. Figure 16 shows the comparison for different event rates. The figure shows that the layered approach is much more scalable. The first reason for the speedup is because, compared to MASSIF, Streaming MASSIF can process events in windows, while MASSIF processes each event one by one. The second reason is that events are filtered in Streaming MASSIF before they are exposed to the expressive reasoning, while each event in MASSIF goes through the expressive reasoning step. The last reason is that the whole background needs to be used for the reasoning step in MASSIF, while in Streaming MASSIF the background is spread between the *Selection* and *Abstraction Module*.

## 7. Discussion

The aim of this research was to design a layered Cascading Reasoning realization that can perform both expressive and temporal reasoning over volatile data streams. The evaluation sections shows that Streaming MASSIF is able to perform high expressive OWL DL reasoning with high throughput. Note that other reasoning approaches exist, such as ASP [41], but we opted for DL since it is a web standard and widely adopted.

### 7.1. Objectives Discussion

Looking back at the *Objectives* set in Section 1, we can now discuss how Streaming MASSIF tackles the various objectives:

1. *Combine various data streams*: Streaming MASSIF can combine various heterogeneous data streams by utilizing a common semantic model, i.e., an ontology, that can be understood throughout the platform. The *Selection Module* allows one to process multiple streams, combining them together, while keeping a common semantics. From these various streams, the *Selection Module* selects those parts that are relevant for further processing.
2. *Integrate background knowledge*: Streaming MASSIF allows the integration with background knowledge both in the *Selection Module* and in the *Abstraction Module*. The integration in the *Selection Module* allows one to combine the data streams with more static data, in order to retrieve more information about the observations in the streams, which typically do not describe the full context they observe. The integration in the *Abstraction Module* allows one to take more context into account to perform the expressive reasoning. The tight coupling between these two modules also allows parts of the static background to be selected in the *Selection Module*, such that it can be used in the *Abstraction Module*.
3. *Integrate complex domain knowledge*: The integration of complex domain knowledge is achieved by allowing expressive reasoning in the *Abstraction Module* in order to correctly interpret the domain. The domain knowledge itself is modeled in the ontology.
4. *Detect temporal dependencies*: Streaming MASSIF can detect temporal dependencies between abstracted events. This is achieved by first abstracting selected observations in the data streams and performing CEP over these abstractions. This allows one to efficiently introduce a temporal aspect in ontology reasoning and integrate complex domain knowledge in CEP. This is more efficient as the direct integration of the temporal domain in DL, i.e., temporal DLs, as they easily become undecidable [18] and CEP is unable to model complex domains [17].
5. *Easy subscription:* Streaming MASSIF allows services to easily subscribe to the data streams, enabling filtering, abstraction, and temporal reasoning, through the use of its unifying query language. This allows services to define their information need in a declarative way, without the need for writing code.

### 7.2. Related Work Comparison

Table 2 compares the related work and the engines used in the evaluation based on the objectives. We also added a column *Service Subscription*, as most engines typically focus on data processing and do not provide mechanisms for service subscription. We note that, even though EP-SPARQL has a query language, the definition of the temporal patterns is complex compared to the pattern definition over abstracted events, as provided by Streaming MASSIF. Furthermore, the reasoning expressivity of EP-SPARQL is low, i.e., RDFS. StreamRule, Ali et al. and CityPulse do not allow the integration of background knowledge when handling the data streams, since they utilize the CQELS RSP engine, which does not allow the integration of static data. Furthermore, their expressive reasoning is done through ASP, while we opted for DL, since it is a web standard and widely adopted. CityPulse also allows the definition of temporal dependencies through CEP; however, the patterns need to be defined programmatically, which further complicate the definition of the information need. Streaming MASSIF integrates CEP and DL reasoning both syntactically, through the use of its unifying query language, and semantically.

Compared to HermiT, RDFox, and TrOWL, Streaming MASSIF is a cascading approach, able to combine the streaming domain with complex and temporal domains, while HermiT, RDFox, and TrOWL focus on performing expressive reasoning on static or slow-moving data. It is clear that Streaming MASSIF targets all objectives.

Table 3 provides an overview of the systems discussed in the related work and how they fit the generalized Cascading Reasoning vision. We see that StreamRule, Ali et al., and CityPulse utilize the CQELS RSP engine for *Stream Processing*. As seen in Table 2, this is the reason they fail to integrate background knowledge in the stream processing, as CQELS is not able to integrate static data. Most of the approaches use annotations to convert data to the common semantic model, while EP-SPARQL is able to rewrite but only from a prolog statement. The inference entailments differ for each platform. Streaming MASSIF is able to achieve the highest expressivity by combining OWL2 DL with CEP. We note that the Allen Algebra utilized in EP-SPARQL for the temporal detections, is typically broader than the temporal patterns allowed in CEP. However, the ontology reasoning supported in EP-SPARQL is low (RDFS) and the definition of the temporal patterns is rather complex compared to Streaming MASSIF.

### 7.3. Evaluation Discussion

In Section 6.1, we evaluated the throughput of different reasoning systems and compared them to a cascading approach. The evaluation shows that, by combining different approaches, both high throughput and high expressivity can be achieved, what is not possible with a monolithic approach. The achieved throughput and expressivity is depending on the components used in the layered approach. Different throughputs can be achieved by combining different engine complexities. For example, if we would combine RSP with RDFox (instead of HermiT), the throughput would be even higher; however, the expressivity would be lower as the expressivity of RDFox is lower as the on of HermiT. Furthermore, higher throughputs can be achieved by duplicating certain components in the layered approach. For example, by distributing multiple RSP engines that each handle different streams and select the relevant parts from their streams, higher throughputs can be achieved. Furthermore, by more intelligently selecting the relevant parts and decreasing the selection rate, the throughput can also be increased, as fewer data need to be processed by the more complex layers.

In the evaluation, we can see that the *Abstraction Module* can easily become the bottleneck with a high number of events, so incremental reasoning techniques should be further researched. Currently, there are no efficient expressive incremental reasoning techniques that also incorporate data property reasoning. We could easily perform the abstraction in parallel and load balance eventsto increase the performance. This is possible in the cases that the events are independent of each other. When multiple physical events should be abstracted together, the query in the lower RSP layer could be adapted to link them together. This would allow one to scale the abstraction module even more since the abstraction of a low number of events is still rather quick, i.e., less than half a second for 30 events. We also note that the higher the selection rate, i.e., the fewer events are selected in the *Selection Module*, the higher the throughput of the complete system, as the abstraction time is still the most time-consuming. It is thus important that only the relevant events can be selected and forwarded.

### 7.4. Streaming MASSIF Limitations & Future Work Directions

One of the limitations of Streaming MASSIF is the fact that the user still manually needs to define a query over all the layers. The query mediation and query rewriting process [42] are currently not researched yet. By enabling the query mediation and rewriting, the query could be defined on a high-level and the query necessary for the selection of the relevant events in the stream could automatically be constructed. This would result in an even easier subscription language.

Another limitation, as discussed above is that the *Abstraction Module* can become the bottleneck when the selection rate is high, i.e., many events still need to be abstracted. As the throughput of the abstraction layer is typically lower than the selection layer, the more data there are that still need to be abstracted, the lower the total throughput will be.

The fact that each layer currently consists of a single engine can be limited as well. As we discussed above, distributing various parts of each layer can further increase the throughput.

In future work, we wish to investigate query mediation and rewriting techniques, such that the query definition can be defined on high-level event definitions and that the parts for the selection over the data streams can be automatically defined. This would further simplify event definitions and service subscriptions. To target the abstraction bottleneck, incremental reasoning techniques or efficient caching techniques need to be further investigated. This would further improve the performance of the expressive reasoning layer and thus improve the total performance of the cascading platform. This is especially necessary when the selection layer is unable to select only a small portion of the stream, and many events need to be abstracted. To further increase the throughput, distribution techniques should be investigated in order to distribute and duplicate various components and layers.

### 7.5. Applicability for Real-World Use-Cases

Table 4 describes some of the sensors and their frequencies in two Smart Cities, i.e., the City of Things in Antwerp (www.imec-int.com/en/cityofthings) and the Aarhus City Lab. (www.smartaarhus.eu/). We see that the frequency of most sensors is typically low and thus Streaming MASSIF can easily process these data streams. Compared to Aarhus, the city of Antwerp transmits all the sensor changes without aggregating them. This means that each Traffic Count sensor transmits the observation of a passing vehicle, while in Aarhus the exact number of measured vehicles since the last transmission is provided. The busier the road, the higher the transmission frequency of the sensor. This shows the strength of Streaming MASSIF and the use of a declarative language. The *Selection Module* utilizes an RSP engine which can easily perform aggregations. The change between the two different types of traffic count sensors would thus only result in the addition of a *Count* statement in the query language. Even if the number of Traffic Count sensors would be very high and they transmit data very frequently, since the data first need to be aggregated, this would result in a very low selection rate, filtering only a very select fragment of aggregated events. The same goes for the Traffic Lights in the smart city of Antwerp. There are eight sensors per intersection transmitting five observations each second. These data typically first need to be aggregated, which results in a very low selection rate; thus, a very small number of events eventually needs to be abstracted. Data from other Smart Cities tell the same story, e.g., in the city of Padova in Italy, data are transmitted once every 10 min [43] by each device. These findings allow us to conclude that Streaming MASSIF can handle cases of real-life smart-city use.

## 8. Conclusions and Future Work

In this paper, we presented Streaming MASSIF, a Cascading Reasoning approach that allows one to perform expressive and temporal reasoning over volatile data streams. Special attention was given to ensure that the platform could *combine various data streams*, *integrate background knowledge*, *integrate complex domain knowledge*, *detect temporal dependencies* and *allow for the easy subscription of services*. In order to tackle these objectives, we propose a cascading reasoning approach, consisting of various layers, each specialized in specific tasks. We defined semantically how these layers collaborate.

Streaming MASSIF is thus a Cascading Reasoning realization consisting of stream processing, continuous information integration, and inference layers. The layers are instantiated by combining RSP, DL reasoning, and CEP to enable expressive reasoning and event processing over high-velocity streams. We described a query languages that combines these various layers, allowing easy querying of the whole reasoning stack without the need to write any code. Our approach can perform expressive reasoning and event processing over high-velocity streams by selecting only the relevant events from the stream.

We have shown that Streaming MASSIF is able to combine high expressive reasoning with a high throughput of processing by combining techniques with different complexities in a layered approach. Furthermore, we have defined on a semantic level how the layers in our cascading cooperate, allowing one to assess the correctness of the approach.

However, when the RSP layer is not able to make this selection from the stream and huge numbers of events need to be abstracted, the platform might become slow. In our future work, we will try to tackle this issue by incorporating load balancing and caching techniques.

We will also investigate query mediation and rewriting to automatically construct the queries on the lower levels, based on the defined concepts on the highest layer. This will further simplify the query definition and bring Stream Reasoning closer to the masses.

## Figures and Tables

**Table 1:** Evaluation of the processing time (in ms) of the different reasoning techniques. By combining very expressive reasoning with very efficient processing, we can achieve high expressivity and high throughput.

Engine/#Events	1	10	100	1000	10,000	25,000	50,000	80,000
RSP	15	15.1	23.1	127.3	398.3	973.5	2011.4	3291.3
RDFox	21.2	21.6	27.7	130.7	500.15	1230.7	2453.3	4405.5
TrOWL	440.3	455.9	415.7	702.8	1292.45	3205.6	7083.3	14,153.0
Hermit	12,895.0	12,972.0	13,440.0	27,885.0	170,532.5			
Cascading	74.2	76.9	74.4	147.5	443.3	1040.0	2303.9	3754.4

**Table 2:** Related work based on the set objectives. (1: not for streams, 2: complex definitions, 3: only programmatically, 4: no unifying subscription language, 5: only incremental changes, 6: using approximations)

	Data Streams	Background Knowledge	Complex Domains	Temporal Dependencies	Unifying QL	Service Subscription
EP-SPARQL [13]	X	/	RDFS	Allen Algebra	X ${}^{2}$	/
StreamRule [19]	X	/ ${}^{1}$	ASP	/	/	/
Ali et al. [21]	X	/ ${}^{1}$	/	ASP	/	/
CityPulse [22]	X	/ ${}^{1}$	ASP	CEP ${}^{3}$	/	X ${}^{4}$
HermiT [15]	/	X	OWL2 DL	/	/	/
RDFox [16]	/ ${}^{5}$	X	OWL2 RL	/	/	/
TrOWL [40]	/ ${}^{5}$	X	OWL2 DL ${}^{6}$	/	/	/
MASSIF [27]	/	X	OWL2 DL	/	/	X
**Streaming** **MASSIF**	**X**	**X**	**OWL2 DL**	**CEP**	**X**	**X**

**Table 3:** Overview of the related work and how they relate to our generalized Cascading Reasoning vision. (1: only programmatically, 2: via CEP rules)

Name	Stream Processing	Continuous Information		Inference Entailment	Unifying QL
		Integration			
EP-SPARQL	Etalis	RSP	Rewriting	RDFS & Allen Algebra	✓
StreamRule	CQELS	RSP	Annotation	ASP	None
Ali et al.	CQELS	RSP	Annotation	Action-Rules in ASP	None
CityPulse	CQELS	RSP	Annotation	ASP & CEP ${}^{1}$	None

**Table 4:** Overview of the produced IoT sensor data in two real-life Smart Cities.

	Frequency	Single Sensor Events/s	# Sensors	Total Events/s
Antwerp				
Air Quality	1 per 30 s	0.034	22	0.667
Temperature	100 per day	0.001	2	0.002
Rain	250 per day	0.003	4	0.012
Traffic Count	±800 per day	±0.01	±115	±1.15
Traffic Lights	5 per second	5	8	40
Aarhus				
Air Quality	1 per 5 min	0.0034	449	1.5
Weather	1 per 5 min	0.0034	9	0.03
Parking	1 per 5 min	0.0034	449	1.5
Traffic Count	3 per hour	0.0008	1	0.008

## References

[1] Al-Fuqaha A., Guizani M., Mohammadi M., Aledhari M., Ayyash M. (2015). Internet of things: A survey on enabling technologies, protocols, and applications. IEEE Commun. Surv. Tutor..

[2] Su X., Riekki J., Nurminen J.K., Nieminen J., Koskimies M. (2015). Adding semantics to internet of things. Concurr. Comput. Pract. Exp..

[3] Della Valle E., Dell’Aglio D., Margara A. (2016). Taming velocity and variety simultaneously in big data with stream reasoning: Tutorial. Proceedings of the 10th ACM International Conference on Distributed and Event-Based Systems.

[4] Ali M.I., Gao F., Mileo A. (2015). Citybench: A configurable benchmark to evaluate rsp engines using smart city datasets. Proceedings of the International Semantic Web Conference.

[5] Barnaghi P., Wang W., Henson C., Taylor K. (2012). Semantics for the Internet of Things: early progress and back to the future. Int. J. Semant. Web Inf. Syst..

[6] Margara A., Urbani J., Van Harmelen F., Bal H. (2014). Streaming the web: Reasoning over dynamic data. Web Semant. Sci. Serv. Agents World Wide Web.

[7] Perera C., Zaslavsky A., Christen P., Georgakopoulos D. (2014). Context aware computing for the internet of things: A survey. IEEE Commun. Surv. Tutor..

[8] Della Valle E., Schlobach S., Krötzsch M., Bozzon A., Ceri S., Horrocks I. (2013). Order matters! harnessing a world of orderings for reasoning over massive data. Semant. Web.

[9] Stuckenschmidt H., Ceri S., Della Valle E., Van Harmelen F. (2010). Towards Expressive Stream Reasoning. Semantic Challenges in Sensor Networks.

[10] Teymourian K. (2014). A Framework for Knowledge-Based Complex Event Processing. Ph.D. Thesis.

[11] Kontchakov R., Zakharyaschev M. (2014). An introduction to description logics and query rewriting. Reasoning Web International Summer School.

[12] Barbieri D.F., Braga D., Ceri S., Valle E.D., Grossniklaus M. (2010). Querying RDF streams with C-SPARQL. SIGMOD Rec..

[13] Anicic D., Fodor P., Rudolph S., Stojanovic N. EP-SPARQL: A unified language for event processing and stream reasoning. Proceedings of the 20th International Conference on World Wide.

[14] Le-Phuoc D., Dao-Tran M., Xavier Parreira J., Hauswirth M., Aroyo L., Welty C., Alani H., Taylor J., Bernstein A., Kagal L., Noy N., Blomqvist E. (2011). A Native and Adaptive Approach for Unified Processing of Linked Streams and Linked Data. Semantic Web—ISWC 2011, Proceedings of the 10th International Semantic Web Conference, Bonn, Germany, 23–27 October 2011.

[15] Shearer R., Motik B., Horrocks I. HermiT: A Highly-Efficient OWL Reasoner. Proceedings of the OWLED 2008.

[16] Nenov Y., Piro R., Motik B., Horrocks I., Wu Z., Banerjee J. (2015). RDFox: A Highly-Scalable RDF Store. Proceedings of the ISWC.

[17] Dell’Aglio D., Della Valle E., van Harmelen F., Bernstein A. (2017). Stream reasoning: A survey and outlook. Data Sci..

[18] Batsakis S., Petrakis E.G., Tachmazidis I., Antoniou G. (2017). Temporal representation and reasoning in OWL 2. Semant. Web..

[19] Mileo A., Abdelrahman A., Policarpio S., Hauswirth M. (2013). Streamrule: a nonmonotonic stream reasoning system for the semantic web. Proceedings of the International Conference on Web Reasoning and Rule Systems.

[20] Gebser M., Leone N., Maratea M., Perri S., Ricca F., Schaub T. Evaluation Techniques and Systems for Answer Set Programming: A Survey. Proceedings of the IJCAI.

[21] Ali M.I., Ono N., Kaysar M., Griffin K., Mileo A. A Semantic Processing Framework for IoT-Enabled Communication Systems. The Semantic Web—ISWC, Proceedings of the International Semantic Web Conference, Bethlehem, PA, USA, 11–15 October 2015.

[22] Puiu D., Barnaghi P., Tonjes R., Kumper D., Ali M.I., Mileo A., Xavier Parreira J., Fischer M., Kolozali S., Farajidavar N. (2016). CityPulse: Large Scale Data Analytics Framework for Smart Cities. IEEE Access.

[23] Taylor K., Leidinger L. (2011). Ontology-Driven Complex Event Processing in Heterogeneous Sensor Networks. The Semanic Web: Research and Applications—ESWC 2011, Proceedings of the Extended Semantic Web Conference, Heraklion, Greece, 29 May–2 June 2011.

[24] Gillani S., Zimmermann A., Picard G., Laforest F. (2017). A query language for semantic complex event processing: Syntax, semantics and implementation. Semant. Web..

[25] Tommasini R., Bonte P., Della Valle E., Mannens E., De Turck F., Ongenae F. (2016). Towards Ontology-Based Event Processing. OWL: Experiences and Directions–Reasoner Evaluation.

[26] Margara A., Cugola G., Collavini D., Dell’Aglio D. (2018). Efficient Temporal Reasoning on Streams of Events with DOTR. The Semantic Web, Proceedings of the European Semantic Web Conference, Heraklion, Greece, 3–7 June 2018.

[27] Bonte P., Ongenae F., De Backere F., Schaballie J., Arndt D., Verstichel S., Mannens E., Van de Walle R., De Turck F. (2017). The MASSIF platform: A modular and semantic platform for the development of flexible IoT services. Knowl. Inf. Syst..

[28] Cugola G., Margara A. (2012). Processing flows of information: From data stream to complex event processing. ACM Comput. Surv..

[29] Luckham D. (2008). The Power of Events: An Introduction to Complex Event Processing in Distributed Enterprise Systems. International Workshop on Rules and Rule Markup Languages for the Semantic Web.

[30] Allen J.F. (1983). Maintaining knowledge about temporal intervals. Commun. ACM.

[31] Dell’Aglio D., Della Valle E., Calbimonte J., Corcho Ó. (2014). RSP-QL Semantics: A Unifying Query Model to Explain Heterogeneity of RDF Stream Processing Systems. Int. J. Semant. Web Inf. Syst..

[32] Grosof B.N., Horrocks I., Volz R., Decker S. (2003). Description logic programs: combining logic programs with description logic. Proceedings of the 12th International Conference on World Wide Web.

[33] Horrocks I., Kutz O., Sattler U. (2006). The Even More Irresistible SROIQ. Kr.

[34] Compton M., Barnaghi P., Bermudez L., GarcíA-Castro R., Corcho O., Cox S., Graybeal J., Hauswirth M., Henson C., Herzog A. (2012). The SSN ontology of the W3C semantic sensor network incubator group. Web Semant. Sci. Serv. Agents World Wide Web..

[35] Barbieri D.F., Braga D., Ceri S., Valle E.D., Huang Y., Tresp V., Rettinger A., Wermser H. (2010). Deductive and Inductive Stream Reasoning for Semantic Social Media Analytics. IEEE Intell. Syst..

[36] Balduini M., Celino I., Dell’Aglio D., Valle E.D., Huang Y., Lee T.K., Kim S., Tresp V. (2014). Reality mining on micropost streams—Deductive and inductive reasoning for personalized and location-based recommendations. Semant. Web..

[37] Tommasini R., Della Valle E. Challenges & Opportunities of RSP-QL Implementations. Proceedings of the WSP/WOMoCoE@ ISWC 2017.

[38] Mauri A., Calbimonte J.P., Dell’Aglio D., Balduini M., Brambilla M., Della Valle E., Aberer K. (2016). Triplewave: Spreading RDF streams on the web. Proceedings of the International Semantic Web Conference.

[39] Dimou A., Vander Sande M., Colpaert P., Verborgh R., Mannens E., Van de Walle R. RML: A Generic Language for Integrated RDF Mappings of Heterogeneous Data. Proceedings of the 7th Workshop on Linked Data on the Web.

[40] Pan J.Z., Ren Y., Jekjantuk N., Garcia J. Reasoning the FMA ontologies with TrOWL. Proceedings of the 2nd International Workshop on OWL Reasoner Evaluation (ORE-2013).

[41] Beck H., Dao-Tran M., Eiter T. (2018). LARS: A Logic-based framework for Analytic Reasoning over Streams. Artif. Intell..

[42] Xiao G., Calvanese D., Kontchakov R., Lembo D., Poggi A., Rosati R., Zakharyaschev M. Ontology-based data access: A survey. Proceedings of the IJCAI.

[43] Zanella A., Bui N., Castellani A., Vangelista L., Zorzi M. (2014). Internet of things for smart cities. IEEE Internet Things J..

